# Dynamic recognition of naloxone, morphine and endomorphin1 in the same pocket of µ-opioid receptors

**DOI:** 10.3389/fmolb.2022.925404

**Published:** 2022-08-16

**Authors:** Xin Zhang, Meng-Yang Sun, Xue Zhang, Chang-Run Guo, Yun-Tao Lei, Wen-Hui Wang, Ying-Zhe Fan, Peng Cao, Chang-Zhu Li, Rui Wang, Xing-Hua Li, Ye Yu, Xiao-Na Yang

**Affiliations:** ^1^ Department of Basic Medicine and Clinical Pharmacy and State Key laboratory of Natural Medicines, China Pharmaceutical University, Nanjing, China; ^2^ Key Laboratory of Preclinical Study for New Drugs of Gansu Province, School of Basic Medical Sciences, Lanzhou University, Lanzhou, China; ^3^ Department of Pharmacology and Chemical Biology, Institute of Medical Sciences, Shanghai Jiaotong University School of Medicine, Shanghai, China; ^4^ Putuo Hospital, Shanghai University of Chinese Traditional Medicine, Shanghai, China; ^5^ Hospital of Integrated Traditional Chinese and Western Medicine, Nanjing University of Chinese Medicine, Nanjing, China; ^6^ State Key Laboratory of Utilization of Woody Oil Resource, Hunan Academy of Forestry, Changsha, China

**Keywords:** morphine, naloxone, endomorphin 1, μ-opioid receptors, ion channel

## Abstract

Morphine, the most widely used analgesic, relieves severe pain by activating the μ-opioid receptor (MOR), whereas naloxone, with only slight structural changes compared to morphine, exhibits inhibitory effect, and is used to treat opioid abuse. The mechanism by which the MOR distinguishes between the two is unclear. Molecular dynamics (MD) simulations on a 1-μs time scale and metadynamics-enhanced conformational sampling are used here to determine the different interactions of these two ligands with MOR: morphine adjusted its pose by continuously flipping deeper into the pocket, whereas naloxone failed to penetrate deeper because its allyl group conflicts with several residues of MOR. The endogenous peptide ligand endomorphin-1 (EM-1) underwent almost no significant conformational changes during the MD simulations. To validate these processes, we employed GIRK4^S143T^, a MOR-activated G_βγ_-protein effector, in combination with mutagenesis and electrophysiological recordings. We verified the role of some key residues in the dynamic recognition of naloxone and morphine and identified the key residue I322, which leads to differential recognition of morphine and naloxone while assisting EM-1 in activating MOR. Reducing the side chain size of I322 (MOR^I322A^) transformed naloxone from an inhibitor directly into an agonist of MOR, and I322A also significantly attenuated the potency of MOR on EM-1, confirming that binding deep in the pocket is critical for the agonistic effect of MOR. This finding reveals a dynamic mechanism for the response of MOR to different ligands and provides a basis for the discovery of new ligands for MOR at the atomic level.

## Introduction

Both morphine and its related µ-opioid receptor (MOR) agonists are used as the most clinically effective drugs for the rapid relief of severe pain (Murphy et al., 2022). High expression in the central nervous system (CNS) and high affinity for opioids have made MOR the most widely studied G-protein-coupled receptors (GPCRs), and numerous *in vitro* and *in vivo* studies have elucidated the mechanisms of MOR response to morphine-induced downstream G-protein signaling pathways at the cellular and physiological levels (Williams et al., 2013; Stein, 2016). As an antagonist of MOR, naloxone is an important tool for rapid reversal of opioid overdose (van Dorp et al., 2007). However, it is worth noting that naloxone is a derivative of morphine and shares the same molecular backbone with it (Marshall Gates, 1956; Kirby, 1967; Vizi et al., 1976), and it is not clear how the MOR receptor recognizes and agonizes or inhibits the corresponding conformational transmission after binding to both.

In recent years, high-resolution crystal structures of MOR bound to the agonist BU72 (Protein Data Bank (PDB) entry: 5C1M) and the antagonist β-FNA (PDB entry: 4DKL) have been reported, demonstrating the different conformations of the binding pocket and transmembrane (TM) helix of MOR in the active and inactive states (Manglik et al., 2012; Huang et al., 2015). The resolution of the structure of the MOR-G protein complex explains the different conformational changes caused by MOR activation (Koehl et al., 2018). The structural analysis of the MOR-G protein complex also explains the conformational changes of the downstream G protein subunits induced by MOR activation (Koehl et al., 2018). Although these structural analyses revealed differences in the conformational changes in G protein activation or inhibition induced by the binding of different ligands to MOR, the details of the conformational changes of the MOR receptor, particularly its binding pocket, upon binding of various ligands, such as morphine, naloxone, and endogenous ligands of MOR (endomorphin-1 or -2), are not well understood.

As a receptor possibly with only a single pocket that could bind small molecules or small peptide (Manglik et al., 2012; Huang et al., 2015; Koehl et al., 2018), the binding of MOR to all ligands seems to be ignored as a fixed pattern, but apparently this is contrary to the ability of MOR proteins to respond to many different ligands, as well as to recognize similar ligands, like morphine and naloxone (only slightly structurally altered) (Zimmerman and Leander, 1990). MOR receptors recognize and respond to different ligands with different modes of binding or action (Valentino and Volkow, 2018), and this distinction between different recognition and response processes need to be studied extensively, as different amino acids in the binding pocket may be involved in or regulate these processes.

**FIGURE 1:**
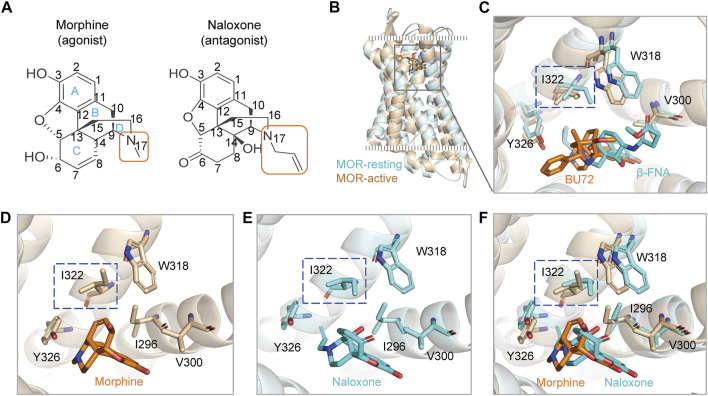
Agonists and antagonists with similar chemical backbones have the same binding pocket in the μ-opioid receptor (MOR). **(A)** chemical structures of morphine and naloxone. Carbon atoms of both compounds are labeled with numbers. **(B)** superimposed structures of rat MOR (rMOR) in the activated (light brown) and inactivated (cyan) states. **(C)** zoom in view of the binding models of BU72 and β-FNA to MOR with reference to the resolved crystal structures. **(D–F)** binding modes of morphine **(D)** and naloxone **(E)** to MOR by using induced fit docking (IFD). The merged model is shown in panel **(F)**. The different conformations of residues I322 in the IFD model for morphine and naloxone are highlighted by the blue dashed boxes.

In this study, by using molecular dynamics (MD) simulations of MOR and ligands on a 1-μs time scale, we explored the allosteric of MOR during binding to different ligands based on recently solved structures of MOR in the activated or resting state, and proposed that three classes of ligands [agonist morphine and antagonist naloxone, which differ only slightly in structure (Figure 1A), and the endogenous peptide agonist endomorphin-1 (EM1)], bind to MOR receptors by different mechanisms. And in combination with mutagenesis, G-protein-induced activation of GIRK4^S143T^ and electrophysiological recordings (He et al., 1999; Hill and Peralta, 2001), we validated the key residues identified by MD simulations and found that one residue (I322) can determine both morphine and naloxone as agonists or inhibitor, and the active potency of EM1 on MOR. Mutation of this amino acid, I322A, could turn naloxone from an antagonist into an agonist of MOR. Our results suggested that the downstream effects induced by ligands at the MOR receptor differ based on subtle changes, which provides some structural basis for understanding the linkage and regulatory mechanisms between MOR-ligand-downstream effects and the design and synthesis of different MOR ligands.

## Results

### Initial conformations of morphine and naloxone binding at MOR extracellular sites change significantly after unbiased MD simulations

MOR provides a single and deep extracellular binding pocket (Manglik et al., 2012; Huang et al., 2015; Koehl et al., 2018) (Figures 1B,C), allowing rapid entry and binding of endogenous or exogenous ligands of different sizes, which may also account for the rapid onset and efficacy of drugs targeting opioid receptors after clinical use. We used a flexible induced fit (IFD) approach to *in silico* dock morphine and naloxone to the extracellular pocket of MOR, and the molecular backbones of both drugs are nearly identical (Figure 1A). The best pose of IFD showed that the initial binding patterns of both are very similar and the residues that interact with them overlapped (Figures 1C–F). However, morphine binds to MOR as an agonist and naloxone binds to MOR as an inhibitor (Stein, 2016), suggesting that the subtle differences in the initial binding of morphine and naloxone trigger the subsequent different allosteric effects of MOR.

**FIGURE 2:**
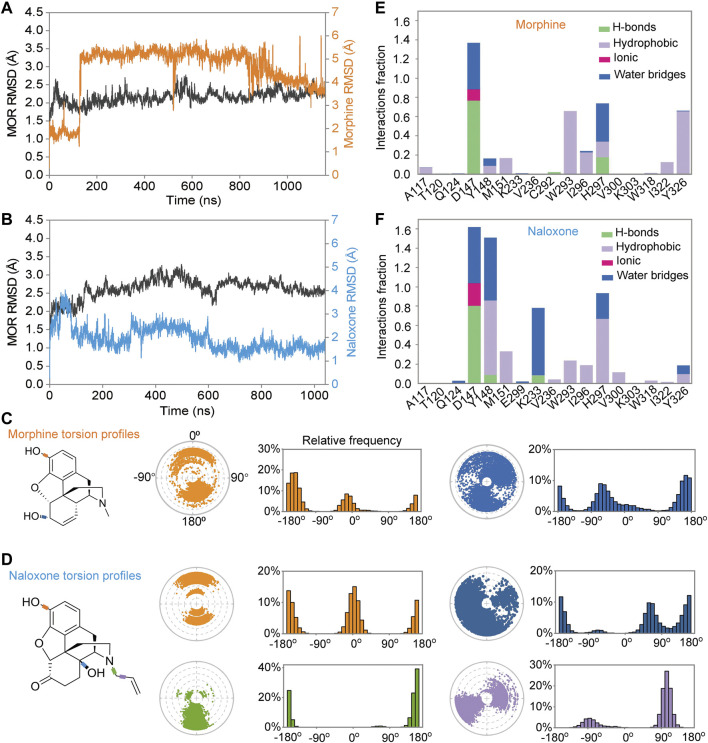
Differences in the binding process of morphine and naloxone to MOR throughout the molecular dynamics (MD) simulations. **(A,B)** backbone root mean square derivation (RMSD) analysis of the binding process of morphine **(A)** and naloxone **(B)** to MOR throughout the MD simulation. **(E,F)** Torsion plots of morphine **(E)** and naloxone **(F)** summarizing the conformational evolution of each rotatable bond every 10 ns (0 ns–1,000 ns) throughout the simulated trajectory. **(C,D)** Two-dimensional schematics of morphine **(C)** and naloxone **(D)** are shown as color-coded rotatable bonds. The radial plots represent the conformation of the torsion bodies throughout the simulation. The center of the radial plot represents the beginning of the simulation, plotting the temporal evolution in the radial direction outward. The histogram summarizes the data on the radial plot, which represents the probability density of the torsion. The relationship between the histogram and the torsional potential gives insight into the conformational strain that the ligand underwent to maintain the rMOR-bound conformation.

To obtain more details of the protein-ligand interaction, we performed unbiased MD simulations of the binding process of morphine and naloxone to the MOR receptor on a 1-µs time scale, using the MOR/naloxone or MOR/morphine IFD models as the initial conformation, respectively. Analysis of the whole simulation process and root mean square derivation (RMSD) analysis revealed that the MOR receptor was in a steady state (first 0.15 µs) during the entry of morphine and naloxone into the binding pocket to reach stable binding, indicating that the simulation system we constructed was in a relatively stable state (Figures 2A,B). Interestingly, morphine showed significant fluctuations around 0.15 µs, indicating that morphine may have undergone a huge pose flip or angle change during the simulation (Figure 2A), while naloxone was relatively stable throughout the 1-µs MD simulation, except for small fluctuations at the beginning (Figure 2B).

In addition, we also analyzed the conformation of the ligands during MD simulations; morphine (Figure 2C) and naloxone (Figure 2D) are shown as two-dimensional schematic diagrams of color-coded rotatable bonds. The radial plots represent the conformation of the torsional body throughout the simulation (Figures 2C,D). The center of the radial plot represents the beginning of the simulation, plotting the temporal evolution in the radial direction outward. The histogram summarizes the data on the radial plot, which represents the probability density of the torsion. The relationship between the histogram and the torsion potential provides insight into the conformational strain experienced by the ligand to maintain the conformation of rMOR binding. Although the hydroxyl group at position-3 is the same for morphine and naloxone (Figure 1A), the torsion angle of this rotational bond for morphine and naloxone showed significant variation throughout the simulation, suggesting that morphine and naloxone have the same molecular backbone, but that minor differences in chemical groups still result in very different binding modes to MOR.

### Dynamic interactions between MOR and morphine/naloxone reveal that I322 is a key residue in the differential recognition of these two ligands by MOR

**FIGURE 3:**
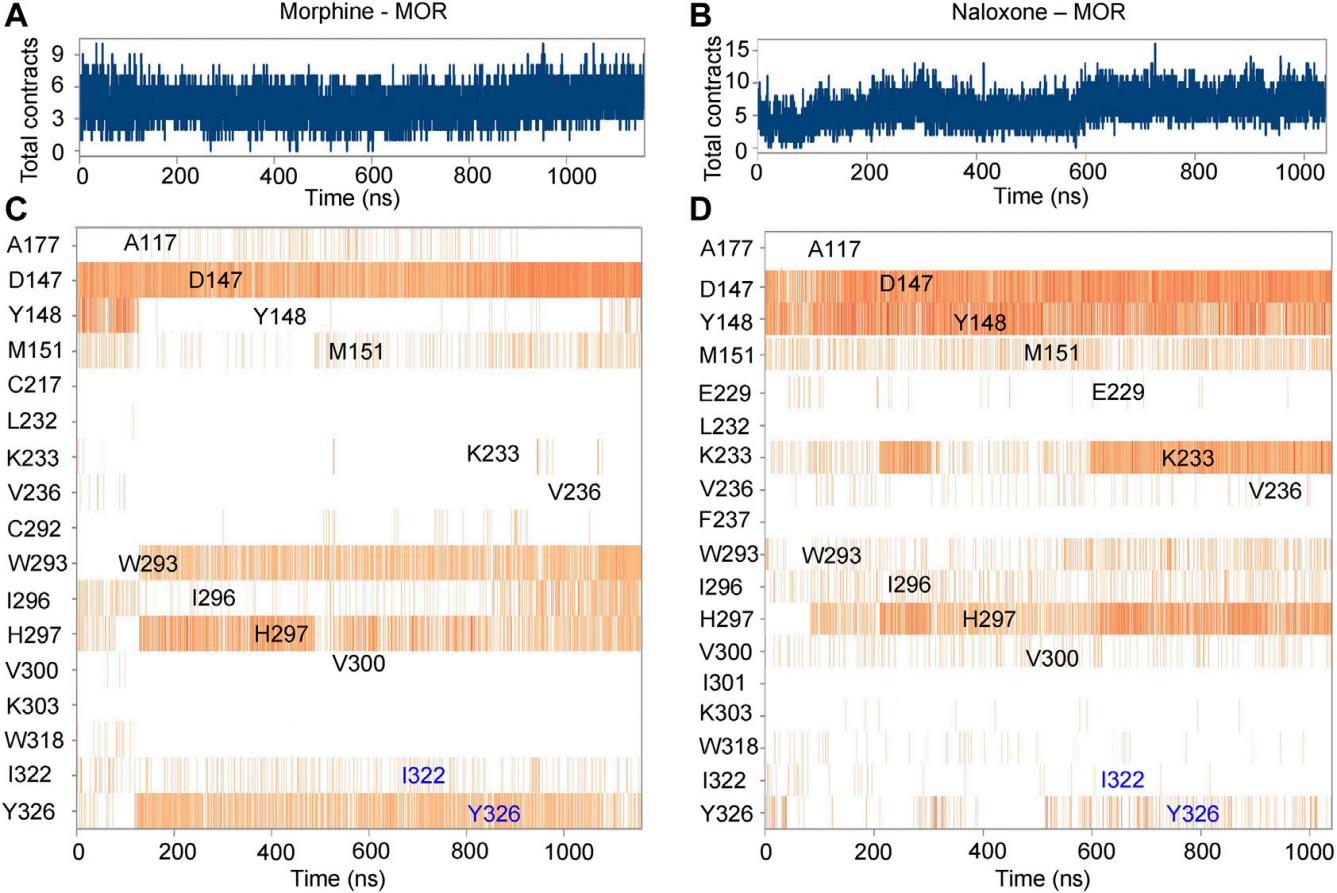
Timeline representation and summary of the interaction between morphine/naloxone and MOR. **(A**,**B)**, time-dependent interactions between MOR and morphine **(A)** or naloxone **(B)** throughout the MD simulations. The top panel represents the total contraction of MOR with the ligand throughout the simulation (one trajectory every 100 ps within 0-1,000 ns). Specific residues interacting with morphine or naloxone are highlighted in blue. The darker orange color indicates the number of residues interacting with the ligand, as some amino acids have multiple specific contracts with the ligand. **(C**,**D)** analysis and summary of the interaction of MOR with morphine **(C)** and naloxone **(D)** throughout the simulation.

Further analysis of the detail of residue interactions in the binding pocket of morphine and naloxone with MOR revealed that D147 interacts tightly and durably with both small molecule ligands; Y148 interacts only in the initial stage of morphine binding, whereas the interaction with naloxone is more persistent (Figures 2E,F, 3). In addition, there were differences in the interaction between the sites of I322 and Y326 during the binding of the two ligands: in contrast to morphine (Figures 2E, 3A,C), naloxone interacted only briefly with these two residues throughout entry and binding (Figures 2F, 3B,D). The differences in binding other sites, such as the E229, K233 and V236 sites, more or less interacted with naloxone (Figures 2F, 3B), whereas morphine did not. These results suggest that although morphine and naloxone are structurally very similar, they bound at different regions in the MOR binding pocket and adopted different binding conformations. These subtle differences in their binding process may ultimately lead to MOR exhibiting agonistic or inhibitory responses.

**FIGURE 4:**
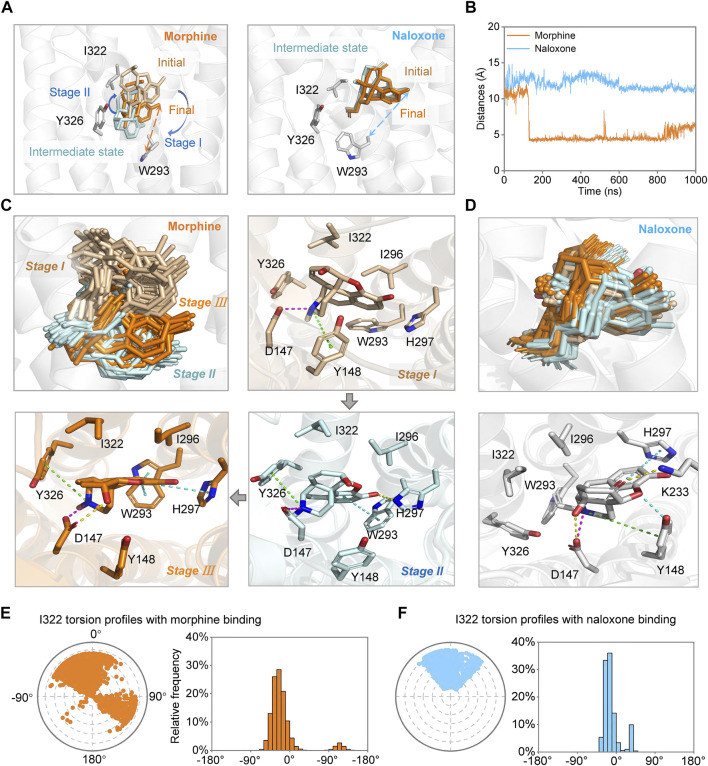
Morphine and naloxone bind dynamically to MOR. **(A)** possible conformational changes of morphine (left) and naloxone (right) at different stages of the binding process. Key residues are indicated by gray sticks and distances between Cα-_W293_ and identical hydroxyl oxygen atom of ligands are indicated by dashed lines of different colors (morphine: orange, naloxone: cyan). **(B)** distance evolutions between Cα-_W293_ and identical hydroxyl oxygen atom of morphine (orange) or naloxone (cyan) over time throughout the MD simulation. **(C**,**D)** conformational changes of morphine **(C)** and naloxone **(D)** and the residues in the binding pocket in MOR at different stages. Key residues during the binding process of the morphine and naloxone at different states are indicated by sticks. **(E**,**F)** the twist diagram of I322 summarizes the conformational evolution of the rotatable bond of I322 upon binding of morphine **(E)** or naloxone **(F)**, including the radial diagram and the bar diagram in the same color. The center of the radial plot represents the beginning of the simulation, plotting the temporal evolution in the radial direction outward. In terms of the range of rotatable angles derived, the range and scale of conformational changes after morphine binding to MOR are much larger than that of naloxone.

Further analysis of the trajectory of ligand movement and conformational changes of MOR throughout the MD simulation revealed significant differences in the dynamic binding of morphine and naloxone to MOR: at the early stage of binding (stage I, Figures 3A,C, 4A,C), morphine interacted with D147 and Y148 sites, then gradually moved away from Y148, approached and interacted with W293 under the attraction of I322 and Y326 (stage II, Figures 3A,C, 4A,C). The fragile interaction between I322 and MOR was disrupted and morphine was pulled deeper by D147 and W293 (stage III, Figures 3A,C, 4A,C). However, the naloxone molecule exhibited a completely different rigidity than morphine, with little flipping or shifting throughout the simulation (Figure 4A right, and Figure 4D) and only some changes in the torsion angle of the chemical groups (Figure 2D). Regardless of the changes and the stage of binding, the allyl of naloxone is like a “boat anchor,” so that the movement of the whole naloxone molecule can only take place around the site I322 (Figures 4A,D). Consistent with this speculation, naloxone binding also significantly constrained the side chain rotation of I322 compared to morphine during MD simulations (Figures 4E,F).

We also measured the distance between the C_α_ atom of W293 and the position-3 hydroxyl O atom of morphine and naloxone (Figure 1A) throughout the binding process, and found that the distance between the morphine and W293 shortened dramatically around 0.15 µs of the simulated binding process (Figure 4B), which may also account for the dramatic fluctuations of the morphine (Figure 2A). On the other hand, naloxone maintained the same W293-C_α_ …O-Naloxone distance throughout (Figure 4B), indicating that the morphine molecule binds deeper than naloxone. Naloxone may not be able to adjust itself to the bottom of the binding pocket where W293 is located, as morphine does, and therefore cannot cause a conformational change in MOR. Such a difference may account for the opposite effects of morphine and naloxone on MOR. The presence of a steric hindrance at the I322, which prevents naloxone from penetrating deeper appears to be the key residue that determines the different binding modes of morphine and naloxone to MOR (see below).

**FIGURE 5:**
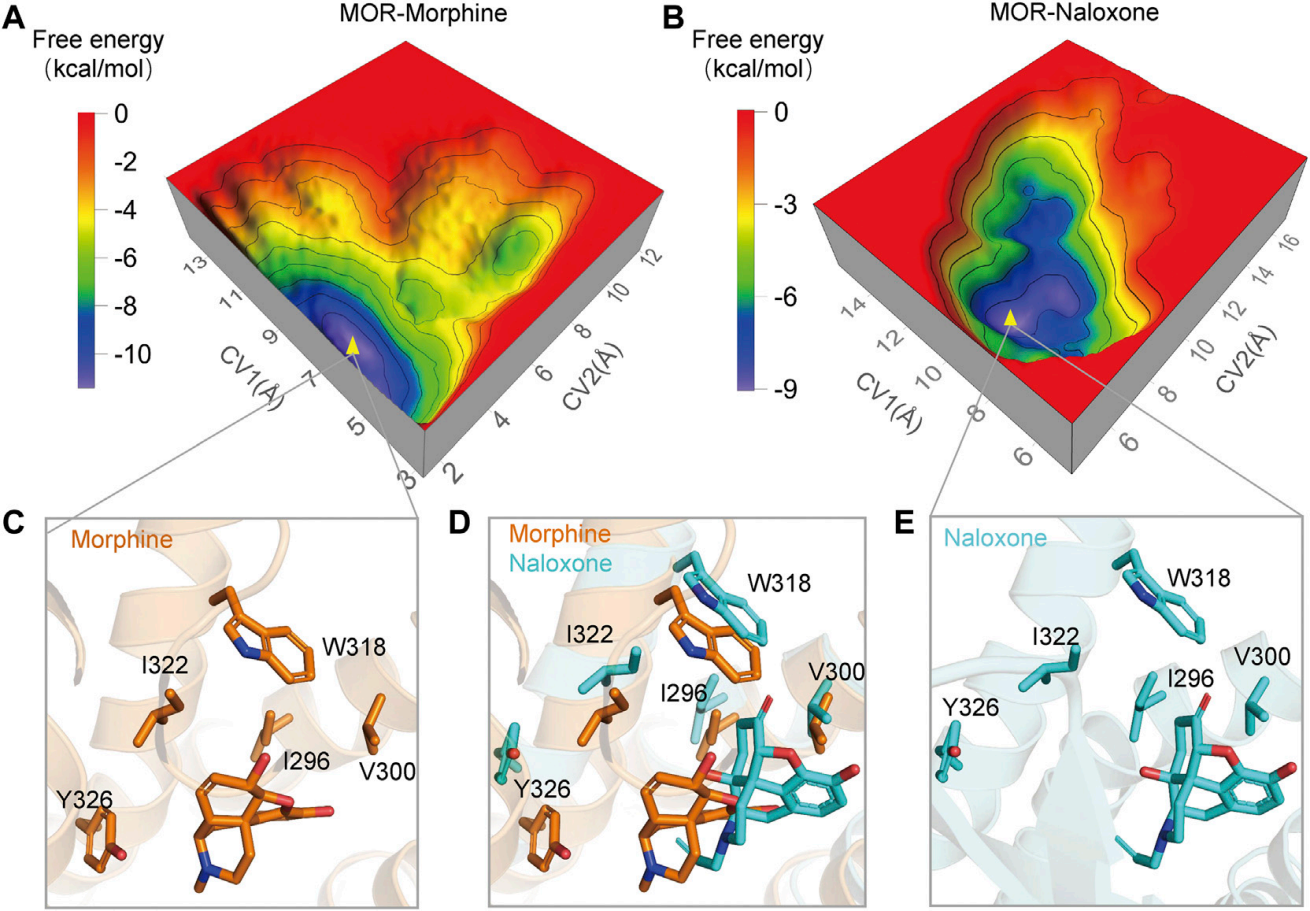
Three-dimensional (3D) free energy reconstruction and optimal binding conformation analysis of MOR/morphine, MOR/naloxone based on metadynamics (MetaD) simulations. **(A,B)** 3D-free energy reconstruction of the interaction between MOR/morphine and MOR/naloxone. **(C**,**E)** optimal conformation of morphine and naloxone binding to MOR during MetaD analysis. **(D)** optimal conformations of morphine and naloxone binding to MOR. Morphine binds more deeply in the MOR pocket compared to naloxone.

In addition, we used metadynamics (MetaD) simulations with enhanced conformational sampling (Limongelli et al., 2010; Söldner et al., 2019) to investigate the interaction between MOR and morphine/naloxone. Three-dimensional (3D) reconstruction of the free energy was performed by defining D1 (the distance between the carbonyl oxygen atom on the side chain of D147 and the N17 atom of morphine or naloxone, Figure 1A) and D2 (distance between Cα atom of I322 and the N17 atom of morphine or naloxone) as the two collective variables (CVs) of MetaD simulations. By extracting the best conformation in the reconstructed 3D-free energy map, the binding of morphine and naloxone in the MOR still showed differences: morphine had a downward shift into a deeper binding pocket compared to the binding position of naloxone (Figure 5), and therefore, the MetaD findings are consistent with those of the MD simulations.

### Combing GIRK4^S143T^ (a MOR-activated G_βγ_ -protein effector), mutagenesis and patch clamp confirms the essential role of key residues in ligand recognition revealed by MD simulations

To be able to easily verify the reliability of MD and MetaD predictions, we introduced the G protein-gated inwardly rectifying potassium channel, GIRK channel, a channel protein that was shown to be an effector of G_βγ_ (Reuveny et al., 1994; Huang et al., 1995; Kofuji et al., 1995; Chan et al., 1997). In general, wild-type (WT) GIRK1/4 is co-expressed to be activated as an effector (Hill and Peralta, 2001). By single point mutation of GRIK4, we obtained the mutant channel GIRK4 (S143T) (He et al., 1999), whose expression alone in cells can respond to activation triggered by MOR agonism-induced G protein dissociation. Therefore, we can use the mutant channel GIRK4^S143T^ as an effector to assess the effect of ligands on MOR, and use electrophysiological recordings to visually monitor the effect of different ligands on MOR. Furthermore, calcium imaging or cAMP assays require high transfection efficiency of WT MOR or its mutants in cell lines (Monory et al., 2000; Baillie et al., 2015; Yudin and Rohacs, 2019), while electrophysiological recordings reduce the difficulty of the assay by simply recording currents on individual expressing cells, and thus can facilitate us performing mutagenesis to verify the MD prediction results.

**FIGURE 6:**
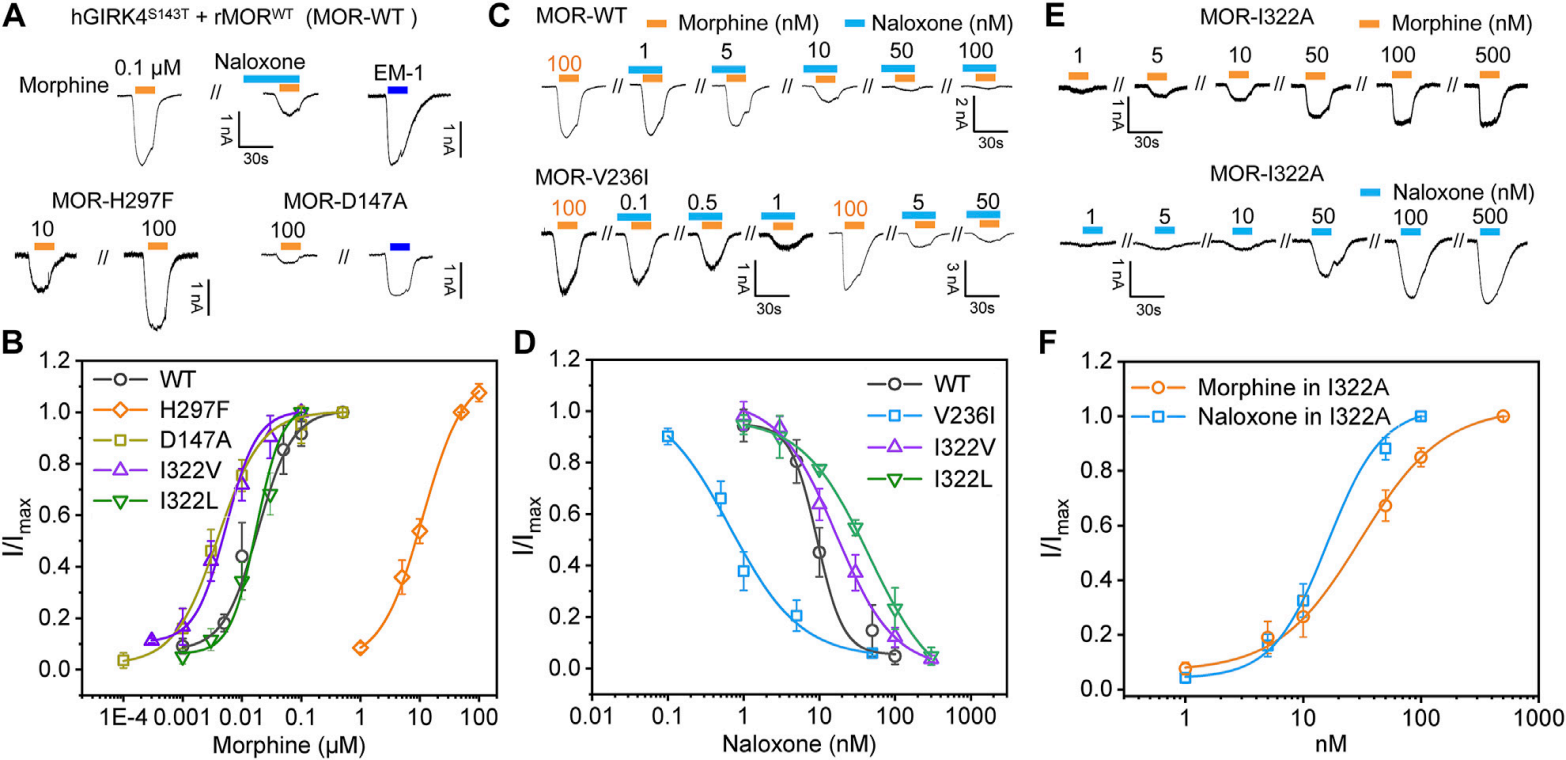
Mutagenesis carried out in key sites located at binding pocket alters the recognition and response of MOR to morphine and naloxone. **(A)** representative current traces for morphine (0.1 μM) and endomorphin-1 (EM-10.1 μM) -induced currents in HEK293 cells with co-expression of hGIRK4^S143T^ channels and wild type (WT) MOR, or MOR mutations, respectively. Morphine-induced currents can be inhibited by naloxone (0.1 μM). **(B)** concentration response relationships for morphine-induced currents in HEK293 cells with co-expression of hGIRK4^S143T^ channels and WT MOR, or MOR mutations, respectively. **(C,D)** representative current traces **(C)** and concentration response relationships **(D)** in cells with co-expression of hGIRK4^S143T^ channels and WT or MOR^V236I^ induced by morphine and naloxone, respectively. **(E,F)** representative current traces **(E)** and concentration response relationships **(F)** in cells with co-expression of hGIRK4^S143T^ channels and WT or MOR^I322A^ induced by morphine or naloxone, respectively. Each point represents the mean ± SE of at least 3 independent measurements. Solid lines are fitted with the *Hill 1* equation for morphine and naloxone-induced currents in WT or MOR^I322A^, respectively.

We co-constructed MOR and GFP into a non-fusion expressing pIRES vector and confirmed the expression of MOR protein in HEK293 cells by fluorescence expression of GFP; in MOR-expressing cells given high extracellular potassium solution perfusion, MOR-expressing cells were considered to also express GIRK4^S143T^ if a large inward potassium current was recorded (Chan et al., 1997; He et al., 1999). In HEK293 cells co-expressing MOR and GIRK4^S143T^, we recorded morphine currents generated by the opening of GIRK4^S143T^ channel induced by WT MOR agonism (Figure 6A). In this paradigm, morphine has a very high affinity for MOR (EC_50_, the concentration yielding half of maximal response = 17.9 nM ± 2.23 nM, Figure 6B), which is also consistent with the affinity for MOR measured by cAMP or calcium imaging assays (Baillie et al., 2015; Yudin and Rohacs, 2019). The use of naloxone, on the other hand, also significantly attenuated the current of the GIRK channel (Figures 6A,C), indicating that our approach of introducing GIRK4^S143T^ to study the ligand function of the opioid receptor is feasible, sensitive, and simpler compared to other methods (Baillie et al., 2015; Yudin and Rohacs, 2019), and suitable for many mutant validations.

Subsequently, we performed mutations at sites with significant interaction with morphine and naloxone according to MD simulations: as key sites for morphine binding and interaction, we selected D147 and H297 for mutation; V236 had weak hydrophobic interaction with naloxone (Figure 3A), but not with morphine (Figure 3A), and was thus instead used to validate the MD of naloxone-MOR interaction. The results confirmed that even 100 μM morphine did weakly activate D147A (Figure 6A); while H297, which is essential for MOR recognition by opioids (Koehl et al., 2018), weakened the affinity of MOR for morphine by ∼600-fold (EC_50_ = 17.9 nM ± 2.23 nM, 11.2 μM ± 1.03 μM for WT and H297F, respectively; Figures 6A,B). Meanwhile, the introduction of a bulkier side chain on V236, V236I (with only one extra methyl group), ∼4-5-fold increased the apparent affinity of MOR by morphine (EC_50_ = 17.9 nM ± 2.23 nM, 3.94 nM ± 0.39 nM for WT and V236I, respectively; Figure 6B), but increased the apparent affinity of the receptor for naloxone by more than 10-fold (IC_50_ = 9.33 nM ± 1.40 nM, 0.76 nM ± 0.20 nM for WT and V236I, respectively; Figures 6C,D, indicating the importance of its hydrophobic interaction with naloxone. Together, these data suggest that the results obtained from the MD and MetaD simulations are reliable and verifiable.

### MOR^I322A^, with reduced side chain size of I322, turned naloxone from an inhibitor directly into an agonist of MOR

MD simulations suggested that the steric hindrance present at I322 prevents deep penetration of naloxone and therefore naloxone cannot activate MOR (Figures 2,3). Also, when analyzing the high-resolution crystal structures of the agonist BU72 (PDB ID: 5C1M) and antagonist β-FNA (PDB ID: 4DKL), bound to MOR, we noticed that the MOR binding pocket in the I322 site of the side chain took a different orientation [(Manglik et al., 2012; Huang et al., 2015) and Figure 1D]. This different conformational change was also observed in *silico* IFD modes of morphine and naloxone (Figures 1A,D—F), which is also in line with our conclusion that there are differences in the action of I322 with morphine and naloxone found when analyzing the results of MD simulations (Figures 2,3).

To further validate this point, the isoleucine at site 322 of MOR was mutated to alanine, I322A, shortening the side chain at this position and reducing the steric hindrance. Such a change did not significantly affect the MOR response and affinity for morphine (EC_50_ = 18.2 ± 3.08 and 17.9 ± 2.23 nM, for I322A and WT MOR, respectively, Figures 6B,E,F). However, MOR^I322A^ reversed the effect of naloxone on MOR: a competitive inhibitor that otherwise inhibits the action of opioid receptors, has agonistic effects like morphine, and is sufficient to cause dissociation of G proteins downstream of opioid receptors (EC_50_ for naloxone in I322A = 29.7 nM ± 2.00 nM; Figures 6E,F).

Those results, combined with the lesser interaction during dynamic recognition of naloxone by MOR (Figure 2), suggest that I322 is not a direct ligand recognition site for morphine and naloxone, but more likely a key amino acid in the pocket shape matching and entry pathway of both molecules. Morphine, due to its smaller size, may be able to overcome the spatial blockade of residues at this position to enter deeper binding sites and trigger downstream G-proteins (Figures 1A, 4B); naloxone, due to the blockade of additional allyl groups by I322 (Figures 1A, 4D), could not penetrate deeper into the MOR to cause conformational changes and interacts more with amino acids in the deep part of the pocket.

To further illustrate this point, we added two additional mutations I332L and I332V (Figure 6). I322L (with comparable steric hindrance of Ile and Leu side chains, but slightly altered spatial alignment) did not significantly alter the apparent affinity of morphine (EC_50_ = 23.1 ± 1.28 and 17.9 nM ± 2.23 nM for I322L and WT MOR, respectively, Figure 6), but decreased the apparent affinity for naloxone by ∼5-fold (IC_50_ = 45.5 ± 1.09 and 9.32 nM ± 1.40 nM for I322L and WT MOR, respectively, Figure 6). I332V (reduces side chain size of one methylene group, thus increasing the accessibility of morphine to deeper pockets) increased the apparent affinity of morphine by ∼3-fold (EC_50_ = 5.37 ± 0.45 and 17.9 nM ± 2.23 nM for I322V and WT MOR, respectively, Figure 6). In contrast, the affinity of naloxone decreased ∼2-fold (IC_50_ = 16.7 ± 2.35 and 9.32 nM ± 1.40 nM for I322L and WT MOR, respectively, Figure 6). Thus, I332 is more in the entry pathway of morphine rather than direct binding.

### I322 is essential for ligand recognition of endomorphin-1, an endogenous peptide ligand of MOR

**FIGURE 7:**
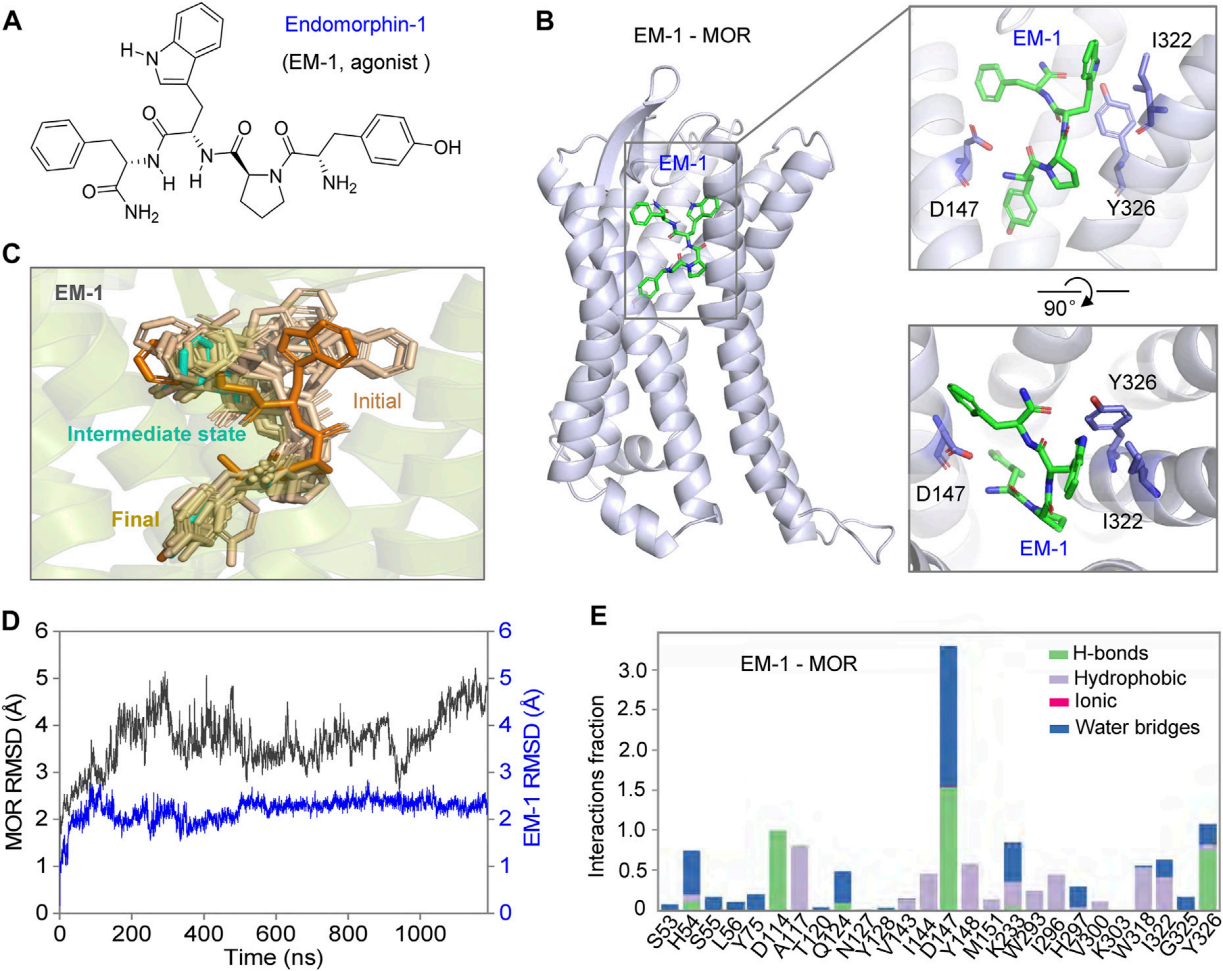
Possible dynamic binding process of EM-1 to MOR. **(A)** chemical structure of EM-1. **(B)** three-dimensional mode of rMOR in complex with EM-1 and zoom-in side- (*upper*) and top views (*bottom*) of the binding pose of EM1-1 during MD simulations. The key sites that interact with EM-1 are shown as sticks for emphasis. **(C)** possible modes of EM-1 binding to MOR. The conformation of EM-1 is shown in different colors at different stages of the binding progress. **(D)** the C_α_-RMSD analysis of MOR and EM-1 throughout the binding progress of MD simulations. **(E)** analysis of the interactions between EM-1 and MOR, divided into four types and shown in different colors.

**FIGURE 8:**
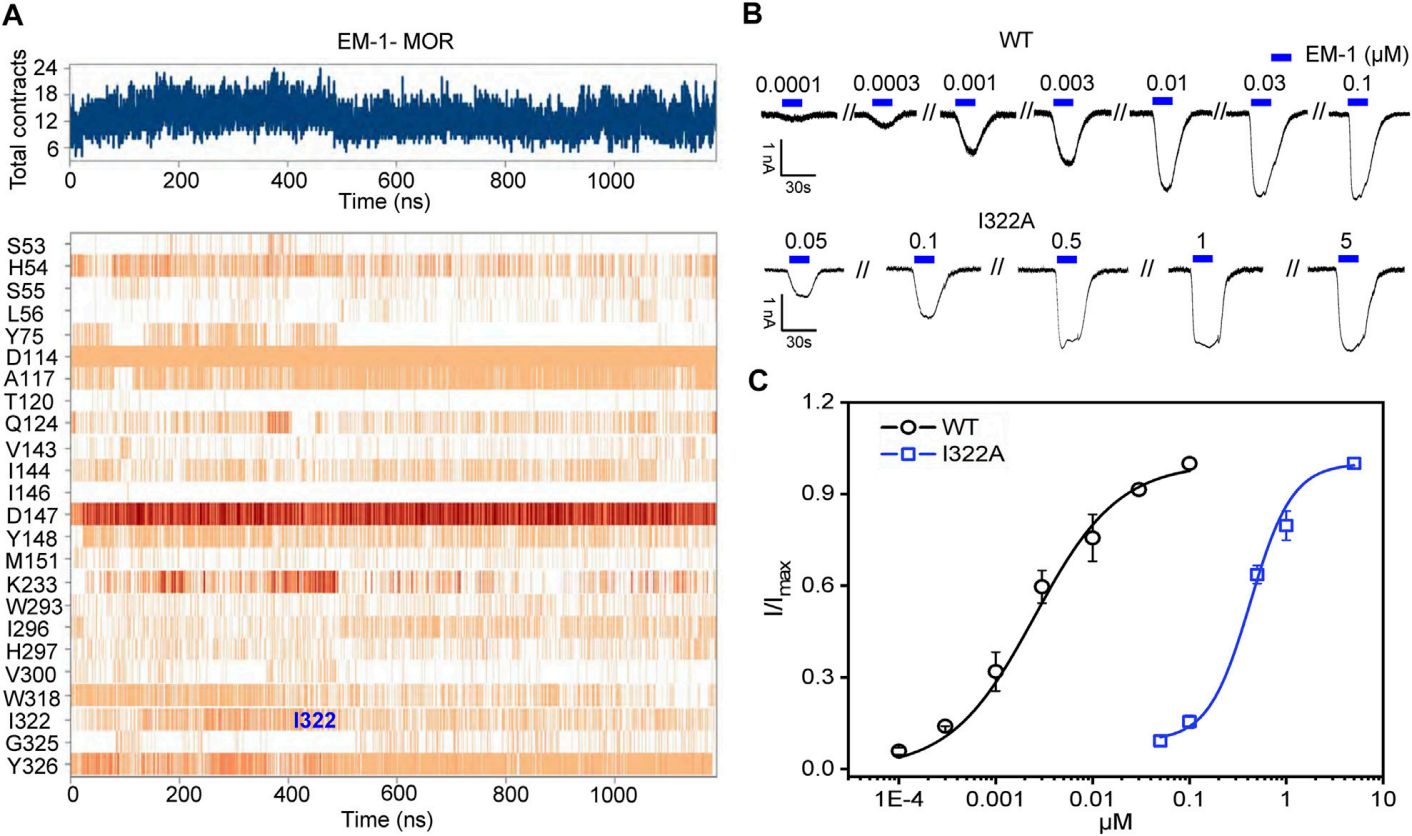
Mutation at site 322 disrupts EM-1 binding to MOR. **(A)** time-dependent interaction between residues of EM-1 and MOR binding pocket. **B**,**C**, representative current traces **(B)** and concentration-response relationships **(C)** for EM-1 in cells with co-expression of hGIRK4^S143T^ channels and WT or MOR^I322A^. Solid lines are fits to EM-1-dependent activation using the *Hill 1* equation. Each point represents the mean ± SE of at least 4 independent measurements.

The binding process and mechanism of action of naloxone and morphine, which are so structurally different, are so different from those of MOR due to a single residue in their binding pocket. So, is the binding of endogenous tetrapeptide, EM-1, to MOR (which is larger compared to naloxone and morphine) influenced by this site (Koehl et al., 2018; Toubia and Khalife, 2019), and is it completely different from the binding of small molecule ligands? To test this idea, we simulated the binding process of EM-1 to MOR using MD simulations. The analysis of the simulated process showed that the EM-1, consisting of four amino acids (Try^1^-Pro^2^-Trp^3^-Phe^4^-NH_2_) (Zadina et al., 1999), bound to MOR and completely occupied the binding cavity of MOR (Figures 7A,B). By assessing the stability of the simulated system throughout the process, it was evident that EM-1 was in a stable state without significant conformational changes during binding to MOR, except for the initiation phase, as with naloxone (Figures 7B–D). Interaction analysis also showed that, unlike the small molecule ligand morphine and naloxone, EM-1 interacted tightly with most residues on the inner surface of the MOR binding cavity (Figures 7E, 8A).

We noted that the I322 position, which plays an important role in the morphine and naloxone binding pathway, may be directly involved in EM-1 binding, and that the I322/EM1 interaction was stronger than morphine/I322 and naloxone/I322 during MD simulations (Figure 8A). Indeed, although MOR^I322A^ was still agonized by EM-1, the apparent affinity for EM-1 was reduced by ∼150-fold (EC_50_ = 387 ± 34.7 and 2.68 nM ± 0.28 nM for I322A and WT MOR, respectively), suggesting that the I322 site directly helps MOR to bind EM-1 more efficiently (Figure 8B), rather than only in the EM1 binding pathway. Thus, naloxone, morphine and EM1 have different dynamic activation processes in the same pocket of the MOR, which is further illustrated by the different effects of MOR^I322A^ on the three aforementioned ligands.

## Discussion

**FIGURE 9:**
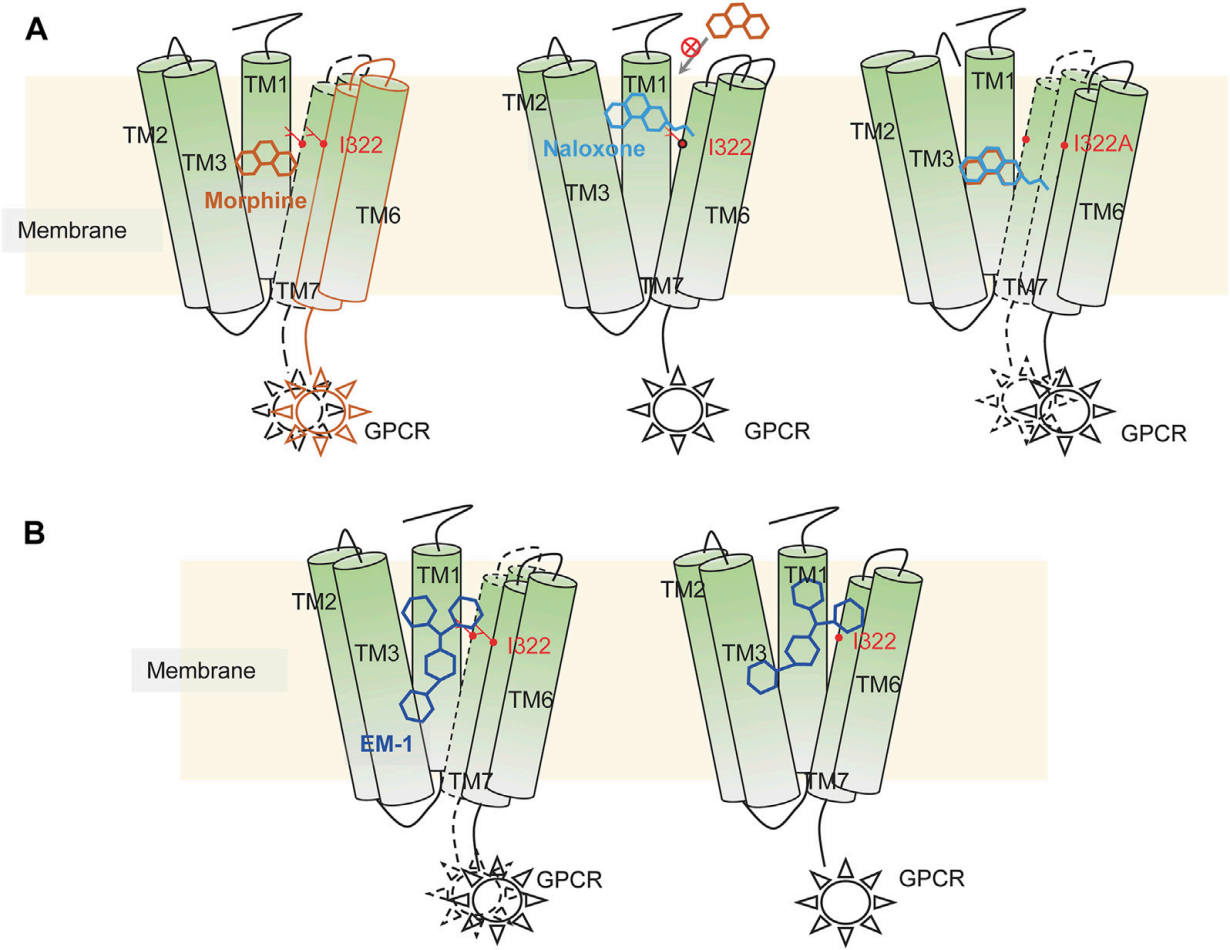
Illustration of possible mechanisms of dynamic recognition of morphine, EM-1 and naloxone by MOR. **(A)** possible mechanisms of MOR recognition of morphine (orange) and naloxone (cyan). Appropriate binding sites located deep within the MOR binding pocket are required for MOR’s full activation, which is critical for the differential effects of morphine and naloxone on MOR. Residues distributed on the inner pocket of MOR prevent naloxone from entering the deeper morphine-binding site due to additional ethylene, which makes naloxone a competitive antagonist of MOR. **(B)** MOR response to EM-1. EM-1 does not readily adjust its conformation into the binding pocket due to its large size, whereas residues on the inner surface of the binding pocket can help EM-1 adopt the proper conformation and rapidly contact with them. Alterations in these residues could disrupt the activation of EM-1 to MOR.

By inducing fitted docking and MD simulations on a 1-μs time scale, confirmed by mutagenesis and electrophysiological recordings, we have identified two classes of MOR ligands with completely different binding modes. The small molecule ligands morphine and naloxone, although sharing an almost identical molecular backbone, have completely opposite effects on MOR. The recognition modes of the two are also completely different: morphine is able to adapt to the shape of the deep MOR binding cavity by flipping itself and changing the bond angles of its chemical groups, eventually forming tight and long-lasting interactions with key sites deep in the binding pocket, such as W293 and Y326. However, after entering the binding cavity, naloxone is unable to penetrate deeper due to the blockade of the side chain of I322 and eventually forms a stable inhibitory conformation with the surrounding amino acids, such as D147, Y148 and K233. Therefore, I322 may be a key site for MOR to recognize and distinguish morphine from naloxone. For the peptide ligand EM1, its larger size makes it impossible to rotate and deflect significantly upon entering the binding cavity of MOR, so it can only be fine-tuned by the transient fragile interaction of amino acid residues on the inner surface of the binding cavity to rapidly form firm interactions with key residues and initiate the conformational transition of MOR. Position I322 may be one such site that helps EM-1 to form a stable conformation. When this “helper” is disrupted, the agonistic effect of EM-1 on MOR remains, but the affinity is greatly reduced (Figure 9).

Chemically, the basic backbone of morphine is a hydrogenated phenanthrene nucleus composed of the A, B, C, D rings, where the phenolic hydroxyl group at the 3-position carbon atom of the A ring, the alcohol hydroxyl group at the 6-position of the C ring and the tertiary amine nitrogen at the 17-position of the D ring are the substitutable groups of morphine (Christrup, 1997; Brook et al., 2017) (Figure 1A). Compounds with phenolic hydroxyl group at position 3 and alcoholic hydroxyl group at position 6 substituted by other groups with similar or higher MOR affinity than morphine as well as better analgesic effect and higher addiction probability, such as oxymorphone with hydroxyl group at position 6 substituted by carbonyl group (Weiss, 1955; Kirby, 1967). When the methyl group at the D-ring 17 position is replaced by allyl, the whole compound becomes naloxone (Zimmerman and Leander, 1990) (Figure 1A). It is noteworthy that regardless of the chemical groups at the other two positions, once the methyl group at the N atom of the 17 position is replaced by a larger chemical group, the chemical molecule becomes an antagonist of the opioid receptor (Zimmerman and Leander, 1990). From the above structure-activity relationships, it is clear that individual chemical groups have an important, even critical, influence on the potency of opioids. Combining our experimental and simulation results, it can be found that this effect might be caused by a single site, I322, in the MOR binding pocket (Figures 3–6): the methyl group of morphine located on the 17-tert-amino nitrogen does not have a particularly tight and long-lasting interaction with I322, and the presence of this residue most likely just helps the morphine entry process pose adjustment as well as giving the compound molecule a downward attractive force to help the binding; while in the binding process of naloxone, the allyl at position 17 makes the compound molecule impeded by the side chain of I322 in the process of entering the binding pocket, so that naloxone cannot continue to penetrate deeper and can only form interactions with the residues above the binding site. However, the conformational changes generated by such interactions are not sufficient to cause MOR opening, and at the same time block the pathway of morphine entry, and the whole opioid receptor can only be in a state of inhibition. The analysis of the structure-activity relationships of small molecules, combined with the mechanism of interaction between the MOR and its ligands at the atomic level, provides a new idea for the design, modification and synthesis of drugs targeting MOR: when structurally modifying ligands, in addition to the interaction between the key binding site in the binding pocket and the compound, the distribution of small molecules in the path of entry into the binding site, which has an effect on the regulatory sites that perturb or help the binding process should also be considered.

DAMGO is another highly selective MOR peptide agonist that has been synthesized. The crystal structure of DAMGO co-crystallization with MOR has also been resolved, revealing that the N-terminal of DAMGO binds to MOR and extends its large C-terminal toward the extracellular loops. This binding pocket and conformational changes are the same as that of morphine and BU72 on MOR, while EM-1 expands in the binding pocket and fills up the space (Figure 7B), interacting with almost all the residues from top to bottom of the inner surface of the binding pocket (Figure 8A). In this process, site I322 may assist the ligand in rapidly adjusting to the proper posture of the pocket; mutation of this site significantly reduces the speed and potency of EM-1 activation of MOR (Figure 8B). Therefore, we suspected that peptide ligands adopt different modes of entry and binding action when binding to MOR: peptide ligands with small-sized amino acid side chains in the sequence can reduce their size to enter the binding cavity of MOR by changing their own conformation, while peptide ligands with large-sized amino acid side chains in the sequence are difficult to fold and reduce, which makes them insert into the binding pocket only with a “stereotypical fixed” pose. But to induce an active conformation of MOR, they need to interact with key residues on the inner surface of the binding pocket and fine-tune their pose and position, which is assisted by residues such as I322.

Morphine, naloxone, and EM-1 are the three classes of MOR ligands, representing semi-synthetic agonists, antagonists, and endogenous peptide agonists, respectively, all three of which bind to the orthosteric pocket of MOR (Vizi et al., 1976; Brook et al., 2017; Toubia and Khalife, 2019). The design and synthesis of preferred ligands such as TRV130, PZM21, and SR-17018 also illustrate that subtle changes in the conformation of the MOR binding pocket can cause differences in downstream effects, thus understanding the linkage and regulatory mechanism between MOR-ligand-downstream effects is a key factor in the design of ideal opioid analgesics (Yudin and Rohacs, 2019; Azevedo Neto et al., 2020). Our investigation of the binding mechanisms of different types of ligands to MOR at the atomic level enables a better understanding of the agonistic and regulatory processes of MOR, and provides an idea of biased ligand design (Wootten et al., 2018) and development from the perspective of MOR receptors.

## Materials and methods

### Chemicals and mutagenesis

The plasmid of pIRES-EGFP-rMOR was kindly gifted from Dr. Xu Zhang. The plasmid of pcDNA3.1-hGIRK4 was synthesized by GENEWIZ Biotech company and then we mutated it to S143T. All compounds were purchased from Sigma. A QuikChange mutagenesis kit was used to construct mutants and confirmed by DNA sequencing (Li et al., 2018).

### Cell culture and electrophysiology

As previously described, human embryonic kidney (HEK-293) cells were cultured in DMEM medium adding 1% penicillin/streptomycin (Gibco), 1% glutamate (Gibco) and 10% FBS (PAM) at 37°C in a humidified atmosphere of 5% CO_2_ and 95% air (Zhao et al., 2014; Wang et al., 2017; Sun et al., 2022). The plasmids of rMOR and hGIRK4 (S143T) was transiently transfected in HEK-293 cells with the transfection agent containing two types of solutions (solution A contains 250 mM CaCl_2_ in pure water; solution B contains (in mM) 1.5 Na_2_HPO_4_, 140 NaCl, and 50 HEPES, and pH was adjusted to 6.96). After mixing the plasmid and solution A, the mixture was added dropwise into solution B of the equal volume, and then it was stirred and mixed with the tip of a pipette tip while dripping. The mixture solution was placed stably at room temperature for 3–5 min, and then added into the cell culture dish. After 6 h of transfection, the culture medium of the dish was changed.

As described previously (Zhao et al., 2016; Yang et al., 2017; Sun et al., 2022), all electrophysiological recordings were performed at room temperature (23°C ± 2°C) with Axon 200B on HEK293 cells after at least 24 h transfection. The conventional whole-cell configuration was performed under the voltage clamp with a holding potential of -60 mV. Patch pipettes had a resistance ranging from 3–5 MΩ when filled with intracellular solution containing (in mM) 30°NaCl, 0.5°CaCl_2_·2H_2_O, 10 HEPES, 120 KCl, 1°MgCl_2_·6H_2_O and 5 EGTA with pH adjusted to 7.4 by Tris-base. Throughout the whole-cell recordings, cells were bathed in the standard external solution containing (in mM) 150°NaCl, 5 KCl, 10 glucose, 2°CaCl_2_·2H_2_ O, 10 HEPES and 1°MgCl_2_·6H_2_O with pH being adjusted to 7.4. The high concentration of K^+^ solution contained (mM) 155 KCl, 10 glucose, 2°CaCl_2_·2H_2_O, 10 HEPES and 1°MgCl_2_·6H_2_O with pH being adjusted to 7.4 by KOH, and was used to induce the inward current of hGIRK4(S143T) channel. All agonists and antagonists were dissolved and diluted by the standard external solution, and all antagonists of rMOR were pre-applied for 1 min and then co-applied in the presence of the agonist. Membrane current signals were amplified by a patch clamp amplifier (Axon 200B, Axon Instruments, Foster City, CA) with low-pass filtered at 1 kHz using low-pass Bessel filters and 0.5 of output gain (α) (low-pass filter at 2°kHz and 50 of output gain (α) for single-channel recordings). A Digidata 1440B interface and a computer running the Clampex and Clampfit 10.6 software (Molecular Devices) were used to sample and analyze all currents. A “Y-tube” method was used to perfuse solutions and compounds throughout all electrophysiological recordings.

### Homology modeling, *in silico* docking and MD simulations

Based on the crystal structures of MOR in complex with BU72 and β-FNA separately (PDB entries: 5C1M and 4DKL), the structure of rat MOR at activation and inactivation states was built and optimized with the program MODELLER (Chovancova et al., 2012). The obtained model was checked and validated by ProCheck (Laskowaski et al., 1993). All ligands were prepared by the LigPrep module in DESMOND by using the OPLS 2005 all-atomic force field (William et al., 1996; Kaminski et al., 2001; Banks et al., 2005). The Induce-Fit-Docking (IFD) module of Schrödinger suite with default parameters was used to acquire the structures of MOR with morphine and naloxone, respectively. There were at least 19 conformations of MOR for per ligand were outputted and the poses behave the best score and the proper interaction were selected. The energy-minimized models of MOR/naloxone, MOR/morphine and MOR/EM-1 were used as the initial structures for MD simulations. A large 1-palmitoyl-2-oleoyl-sn-glycero-3-phosphocholine (POPC, 300 K) bilayer, available in System Builder of DESMOND (Dror et al., 2011), was built to generate a suitable membrane system based on the OPM database (https://opm.phar.umich.edu) (Lomize et al., 2012), in which the TM domain of the MOR could be embedded properly. The MOR/naloxone/POPC, MOR/morphine/POPC, and MOR/naloxone/POPC system was dissolved in simple point charge (SPC) water molecules. Counter ions were then added to compensate for the net negative charge of the system. NaCl (150 mM) was added into the simulation box that represents background salt at physiological condition. The DESMOND default relaxation protocol was applied to each system prior to the simulation run. 1) 100 ps simulations in the NVT (constant number (N), volume (V), and temperature (T)) ensemble with Brownian kinetics using a temperature of 10 K with solute heavy atoms constrained; 2) 12 ps simulations in the NVT ensemble using a Berendsen thermostat with a temperature of 10 K and small-time steps with solute heavy atoms constrained; 3) 12 ps simulations in the NPT (constant number (N), pressure (P), and temperature (T)) ensemble using a Berendsen thermostat and barostat for 12 ps simulations at 10 K and 1 atm, with solute heavy atoms constrained; 4) 12 ps simulations in the NPT ensemble using a Berendsen thermostat and barostat at 300 K and 1 atm with solute heavy atoms constrained; 5) 24 ps simulations in the NPT ensemble using a Berendsen thermostat and barostat at 300 K and 1 atm without constraint. After equilibration, the MD simulations were performed for ∼1-µs. Long-range electrostatic interactions were computed using a smooth Particle Mesh Ewald method. The trajectory recording interval was set to 200-ps and other default parameters of DESMOND were used during MD simulation runs. All simulations used the all-atomic OPLS_2005 force field (William et al., 1996; Kaminski et al., 2001; Banks et al., 2005), which was used for proteins, ions, lipids and the SPC waters. The Simulation Interaction Diagram (SID) module in DESMOND was used for exploring the interaction analysis between morphine/naloxone/EM1 and MOR.

All MD simulations were performed in DELL T7920 armed by NVIDIA TESLTA K40C or CAOWEI 4028GR armed by NVIDIA TESLTA K80. The simulation system preparation, trajectory analysis and visualization were performed on DELL M3800 graphic working stations. The distance between residues and ligands was measured in the simulation event analysis module of DESMOND (Zhao et al., 2014; Sun et al., 2022).

### Metadynamics simulations

As previously described (Laio et al., 2005; Limongelli et al., 2010; Zhao et al., 2014; Wang et al., 2018; Yang et al., 2021), all metadynamics simulations were performed by DESMOND under NPT and periodic boundary conditions using the default parameters at constant temperature (300 K) and pressure (1 bar). The distances between the key residue of MOR and the ligand (morphine and naloxone) were set to CVs. D1 (the distance between the carbonyl oxygen atom on the side chain of D147 and the N17 atom of morphine or naloxone, Figure 1A) and D2 (distance between Cα atom of I322 and the N17 atom of morphine or naloxone ) was defined as two collective variables (CVs) of MetaD simulations. The parameters for height, width of the Gaussian, and the interval were set to 0.03 kcal/mol, 0.05 Å and 0.09 ps, respectively. The DESMOND default relaxation protocol was applied to each system prior to the MetaD simulation run (same steps as for conventional molecular dynamics, see above). All metadynamics simulations run 60 ns until they showed free diffusion along the defined CV. The sum of the Gaussians and the free-energy surface were generated by METADYNAMICS ANALYSIS tools of DESMOMD. The bias *V(s, t)* is typically constructed in the form of periodically added repulsive Gaussians, where *s* is the chosen CV which could be multidimensional. Therefore, the free-energy surface (FES) can be constructed in the space spanned by those CVs. The bias potential *V (s, t)* at time *t* can be written as (Gervasio et al., 2005; Clark et al., 2016; Xu et al., 2021):

$$V\left(s,t\right)=\overset{t}{\underset{0}{\int }}\omega exp\left(-\overset{d}{\underset{i=1}{\sum }}\frac{{\left(S_{i}-S_{i}\left(t^{\prime }\right)\right)}^{2}}{2\sigma_{i}^{2}}\right)\text{d}t^{\prime }$$

where ω is the Gaussian height controlled by the deposition stride, $S_{i}$ is one of *d* CVs, and *σ*_*i*_ is the Gaussian width. This method pushes the system to escape the local minima to find the nearest saddle point on the FES. When the transient happens, the bias provides the free-energy estimate as:

V(s,t)= −F(S)+C

where C is an arbitrary additive constant. Since the absolute free energy is normally not important, this constant can be readily eliminated for calculating the free-energy difference.

### Data analysis

All electrophysiological recordings were analyzed using Clampfit 10.6 (Molecular Devices). Concentration-response relationships of rMOR WT and mutations were obtained by measuring currents in response to different concentrations of morphine, naloxone and endomorphin-1, and all results that were used to generate a concentration-response relationship were from the same group. The data were fit to the Hill1 equation: *I*/*I*_max_ = 1/{1+[EC_50_/( morphine, naloxone or endomorphin-1)] ^n^}, where *I* is the normalized current at a given concentration of ligands, *I*_max_ is the maximum normalized current, EC_50_ is the morphine, naloxone or EM-1 concentration producing half of the maximum current, and *n* is the Hill1 coefficient.

## Data Availability

The original contributions presented in the study are included in the article/supplementary material, further inquiries can be directed to the corresponding authors.

## References

[B1] Azevedo Neto J. Costanzini A. De Giorgio R. Lambert D. G. Ruzza C. Calo G. (2020). Biased versus partial agonism in the search for safer opioid analgesics. Molecules 25 (17), E3870. 10.3390/molecules25173870 32854452 PMC7504468

[B2] Baillie L. D. Schmidhammer H. Mulligan S. J. (2015). Peripheral mu-opioid receptor mediated inhibition of calcium signaling and action potential-evoked calcium fluorescent transients in primary afferent CGRP nociceptive terminals. Neuropharmacology 93, 267–273. 10.1016/j.neuropharm.2015.02.011 25721395

[B3] Banks J. L. Beard H. S. Cao Y. Cho A. E. Damm W. Farid R. (2005). Integrated modeling program, applied chemical theory (IMPACT). J. Comput. Chem. 26 (16), 1752–1780. 10.1002/jcc.20292 16211539 PMC2742605

[B4] Brook K. Bennett J. Desai S. P. (2017). The chemical history of morphine: An 8000-year journey, from resin to de-novo synthesis. J. Anesth. Hist. 3 (2), 50–55. 10.1016/j.janh.2017.02.001 28641826

[B5] Chan K. W. Sui J. L. Vivaudou M. Logothetis D. E. (1997). Specific regions of heteromeric subunits involved in enhancement of G protein-gated K+ channel activity. J. Biol. Chem. 272 (10), 6548–6555. 10.1074/jbc.272.10.6548 9045681

[B6] Chovancova E. Pavelka A. Benes P. Strnad O. Brezovsky J. Kozlikova B. (2012). Caver 3.0: a tool for the analysis of transport pathways in dynamic protein structures. PLoS Comput. Biol. 8 (10), e1002708. 10.1371/journal.pcbi.1002708 23093919 PMC3475669

[B7] Christrup L. L. (1997). Morphine metabolites. Acta Anaesthesiol. Scand. 41 (1 Pt 2), 116–122. 10.1111/j.1399-6576.1997.tb04625.x 9061094

[B8] Clark A. J. Tiwary P. Borrelli K. Feng S. Miller E. B. Abel R. (2016). Prediction of protein-ligand binding poses via a combination of induced fit docking and metadynamics simulations. J. Chem. Theory Comput. 12 (6), 2990–2998. 10.1021/acs.jctc.6b00201 27145262

[B9] Dror R. O. Arlow D. H. Maragakis P. Mildorf T. J. Pan A. C. Xu H. (2011). Activation mechanism of the β2-adrenergic receptor. Proc. Natl. Acad. Sci. U. S. A. 108 (46), 18684–18689. 10.1073/pnas.1110499108 22031696 PMC3219117

[B10] Gervasio F. L. Laio A. Parrinello M. (2005). Flexible docking in solution using metadynamics. J. Am. Chem. Soc. 127 (8), 2600–2607. 10.1021/ja0445950 15725015

[B11] He C. Zhang H. Mirshahi T. Logothetis D. E. (1999). Identification of a potassium channel site that interacts with G protein betagamma subunits to mediate agonist-induced signaling. J. Biol. Chem. 274 (18), 12517–12524. 10.1074/jbc.274.18.12517 10212228

[B12] Hill J. J. Peralta E. G. (2001). Inhibition of a Gi-activated potassium channel (GIRK1/4) by the Gq-coupled m1 muscarinic acetylcholine receptor. J. Biol. Chem. 276 (8), 5505–5510. 10.1074/jbc.M008213200 11060307

[B13] Huang C. L. Slesinger P. A. Casey P. J. Jan Y. N. Jan L. Y. (1995). Evidence that direct binding of G beta gamma to the GIRK1 G protein-gated inwardly rectifying K+ channel is important for channel activation. Neuron 15 (5), 1133–1143. 10.1016/0896-6273(95)90101-9 7576656

[B14] Huang W. Manglik A. Venkatakrishnan A. J. Laeremans T. Feinberg E. N. Sanborn A. L. (2015). Structural insights into µ-opioid receptor activation. Nature 524 (7565), 315–321. 10.1038/nature14886 26245379 PMC4639397

[B15] Kaminski G. A. Friesner R. A. Tirado-Rives J. Jorgensen W. L. (2001). Evaluation and reparametrization of the OPLS-AA force field for proteins via comparison with accurate quantum chemical calculations on peptides. J. Phys. Chem. B 105, 6474–6487. 10.1021/jp003919d

[B16] Kirby G. W. (1967). Biosynthesis of the morphine alkaloids. Science 155 (3759), 170–173. 10.1126/science.155.3759.170 5332945

[B17] Koehl A. Hu H. Maeda S. Zhang Y. Qu Q. Paggi J. M. (2018). Structure of the mu-opioid receptor-Giprotein complex. Nature 558 (7711), 547–552. 10.1038/s41586-018-0219-7 29899455 PMC6317904

[B18] Kofuji P. Davidson N. Lester H. A. (1995). Evidence that neuronal G-protein-gated inwardly rectifying K+ channels are activated by G beta gamma subunits and function as heteromultimers. Proc. Natl. Acad. Sci. U. S. A. 92 (14), 6542–6546. 10.1073/pnas.92.14.6542 7604029 PMC41554

[B19] Laio A. Rodriguez-Fortea A. Gervasio F. L. Ceccarelli M. Parrinello M. (2005). Assessing the accuracy of metadynamics. J. Phys. Chem. B 109 (14), 6714–6721. 10.1021/jp045424k 16851755

[B20] Laskowaski R. A. Macarthur M. W. Doss D. S. Thornton J. (1993). PROCHECK: a program to check the stereochemicai quality of protein structures. J. Appl. Cryst. 26, 283–291. 10.1107/S0021889892009944

[B21] Li B. Wang J. Cheng X. Liu Y. Yang Y. Yang X. (2018). Molecular mechanism underlying the subtype-selectivity of competitive inhibitor NF110 and its distinct potencies in human and rat P2X3 receptors. Sci. Bull. 63 (24), 1616–1625. 10.1016/j.scib.2018.11.016 36658853

[B22] Limongelli V. Bonomi M. Marinelli L. Gervasio F. L. Cavalli A. Novellino E. (2010). Molecular basis of cyclooxygenase enzymes (COXs) selective inhibition. Proc. Natl. Acad. Sci. U. S. A. 107 (12), 5411–5416. 10.1073/pnas.0913377107 20215464 PMC2851773

[B23] Lomize M. A. Pogozheva I. D. Joo H. Mosberg H. I. Lomize A. L. (2012). OPM database and PPM web server: resources for positioning of proteins in membranes. Nucleic Acids Res. 40 (Database issue), D370–D376. 10.1093/nar/gkr703 21890895 PMC3245162

[B24] Manglik A. Kruse A. C. Kobilka T. S. Thian F. S. Mathiesen J. M. Sunahara R. K. (2012). Crystal structure of the μ-opioid receptor bound to a morphinan antagonist. Nature 485 (7398), 321–326. 10.1038/nature10954 22437502 PMC3523197

[B25] Marshall Gates G. T. Tschudi G. (1956). The synthesis of morphine. J. Am. Chem. Soc. 78, 1380–1393. 10.1021/ja01588a033

[B26] Monory K. Bourin M. C. Spetea M. Tomboly C. Toth G. Matthes H. W. (2000). Specific activation of the mu opioid receptor (MOR) by endomorphin 1 and endomorphin 2. Eur. J. Neurosci. 12 (2), 577–584. 10.1046/j.1460-9568.2000.00936.x 10712637

[B27] Murphy P. B. Bechmann S. Barrett M. J. (2022). “Morphine,” in StatPearls (Treasure Island (FL).

[B28] Reuveny E. Slesinger P. A. Inglese J. Morales J. M. Iniguez-Lluhi J. A. Lefkowitz R. J. (1994). Activation of the cloned muscarinic potassium channel by G protein beta gamma subunits. Nature 370 (6485), 143–146. 10.1038/370143a0 8022483

[B29] Söldner C. A. Horn A. H. C. Sticht H. (2019). A metadynamics-based protocol for the determination of GPCR-ligand binding modes. Int. J. Mol. Sci. 20 (8), E1970. 10.3390/ijms20081970 31013635 PMC6514967

[B30] Stein C. (2016). Opioid receptors. Annu. Rev. Med. 67, 433–451. 10.1146/annurev-med-062613-093100 26332001

[B49] Sun M. Y. Zhang X. Yu P. C. Liu D. Yang Y. Cui W. W. (2022). Vanilloid agonist-mediated activation of TRPV1 channels requires coordinated movement of the S1–S4 bundle rather than a quiescent state. Sci. Bull. 67 (10), 1062–1076. 10.1016/j.scib.2022.02.016 36546250

[B31] Toubia T. Khalife T. (2019). The endogenous opioid system: role and dysfunction caused by opioid therapy. Clin. Obstet. Gynecol. 62 (1), 3–10. 10.1097/GRF.0000000000000409 30398979

[B32] Valentino R. J. Volkow N. D. (2018). Untangling the complexity of opioid receptor function. Neuropsychopharmacology 43 (13), 2514–2520. 10.1038/s41386-018-0225-3 30250308 PMC6224460

[B33] van Dorp E. Yassen A. Dahan A. (2007). Naloxone treatment in opioid addiction: the risks and benefits. Expert Opin. Drug Saf. 6 (2), 125–132. 10.1517/14740338.6.2.125 17367258

[B34] Vizi E. S. Foldes F. F. Rich J. Knoll J. (1976). The structure-action relationship and kinetics of some naloxone and naltrexone derivatives. Pharmacology 14 (1), 76–85. 10.1159/000136582 959310

[B35] Wang J. Sun L. F. Cui W. W. Zhao W. S. Ma X. F. Li B. (2017). Intersubunit physical couplings fostered by the left flipper domain facilitate channel opening of P2X4 receptors. J. Biol. Chem. 292 (18), 7619–7635. 10.1074/jbc.M116.771121 28302727 PMC5418059

[B36] Wang J. Wang Y. Cui W. W. Huang Y. Yang Y. Liu Y. (2018). Druggable negative allosteric site of P2X3 receptors. Proc. Natl. Acad. Sci. U. S. A. 115 (19), 4939–4944. 10.1073/pnas.1800907115 29674445 PMC5948998

[B37] Weiss U. (1955). Derivatives of morphine. I. 14-Hydroxydihydromorphinone. J. Am. Chem. Soc. 77 (22), 5891–5892. 10.1021/ja01627a033

[B38] William L. Jorgensen S. Maxwell D. Tirado-Rives J. (1996). Development and testing of the OPLS all-atom force field on conformational energetics and properties of organic liquids. J. Am. Chem. Soc. 118, 11225–11236. 10.1021/ja9621760

[B39] Williams J. T. Ingram S. L. Henderson G. Chavkin C. von Zastrow M. Schulz S. (2013). Regulation of mu-opioid receptors: desensitization, phosphorylation, internalization, and tolerance. Pharmacol. Rev. 65 (1), 223–254. 10.1124/pr.112.005942 23321159 PMC3565916

[B40] Wootten D. Christopoulos A. Marti-Solano M. Babu M. M. Sexton P. M. (2018). Mechanisms of signalling and biased agonism in G protein-coupled receptors. Nat. Rev. Mol. Cell Biol. 19 (10), 638–653. 10.1038/s41580-018-0049-3 30104700

[B41] Xu J. Cao X. M. Hu P. (2021). Accelerating metadynamics-based free-energy calculations with adaptive machine learning potentials. J. Chem. Theory Comput. 17 (7), 4465–4476. 10.1021/acs.jctc.1c00261 34100605

[B42] Yang X. N. Niu Y. Y. Liu Y. Yang Y. Wang J. Cheng X. Y. (2017). The nonproton ligand of acid-sensing ion channel 3 activates mollusk-specific FaNaC channels via a mechanism independent of the native FMRFamide peptide. J. Biol. Chem. 292 (52), 21662–21675. 10.1074/jbc.M117.814707 29123030 PMC5766947

[B43] Yang P. L. Li X. H. Wang J. Ma X. F. Zhou B. Y. Jiao Y. F. (2021). GSK1702934A and M085 directly activate TRPC6 via a mechanism of stimulating the extracellular cavity formed by the pore helix and transmembrane helix S6. J. Biol. Chem. 297 (4), 101125. 10.1016/j.jbc.2021.101125 34461094 PMC8458982

[B44] Yudin Y. Rohacs T. (2019). The G-protein-biased agents PZM21 and TRV130 are partial agonists of mu-opioid receptor-mediated signalling to ion channels. Br. J. Pharmacol. 176 (17), 3110–3125. 10.1111/bph.14702 31074038 PMC6692666

[B45] Zadina J. E. Martin-Schild S. Gerall A. A. Kastin A. J. Hackler L. Ge L. J. (1999). Endomorphins: novel endogenous mu-opiate receptor agonists in regions of high mu-opiate receptor density. Ann. N. Y. Acad. Sci. 897, 136–144. 10.1111/j.1749-6632.1999.tb07885.x 10676442

[B46] Zhao W. S. Wang J. Ma X. J. Yang Y. Liu Y. Huang L. D. (2014). Relative motions between left flipper and dorsal fin domains favour P2X4 receptor activation. Nat. Commun. 5, 4189. 10.1038/ncomms5189 24943126

[B47] Zhao W. S. Sun M. Y. Sun L. F. Liu Y. Yang Y. Huang L. D. (2016). A highly conserved salt bridge stabilizes the kinked conformation of β2, 3-sheet essential for channel function of P2X4 receptors. J. Biol. Chem. 291 (15), 7990–8003. 10.1074/jbc.M115.711127 26865631 PMC4825005

[B48] Zimmerman D. M. Leander J. D. (1990). Opioid antagonists: structure activity relationships. NIDA Res. Monogr. 96, 50–60. 2172827

